# Supplementary material for: Saccharomyces boulardii (CNCM I-745) improves intestinal damage in sepsis by remodeling intestinal flora structure
Source: Front Cell Infect Microbiol. 2025 Jul 31;15:1602792. doi: 10.3389/fcimb.2025.1602792 (PMC12350329; doi:10.3389/fcimb.2025.1602792)
Supplement: Supplementary file 1 [file DataSheet1.zip › summary/summary/6_Taxonomy_community/6_Krona.html]

Javascript must be enabled to view this page.

members
magnitude
magnitudeUnassigned

DW4\_1
DW4\_2
DW4\_3
DW4\_4
DW4\_5
DW4\_6
DW0\_1
DW0\_2
DW0\_3
DW0\_4
DW0\_5
DW0\_6
CW4\_1
CW4\_2
CW4\_3
CW4\_4
CW4\_5
CW4\_6
CW0\_1
CW0\_2
CW0\_3
CW0\_4
CW0\_5
CW0\_6
BW4\_1
BW4\_2
BW4\_3
BW4\_4
BW4\_5
BW4\_6
BW0\_1
BW0\_2
BW0\_3
BW0\_4
BW0\_5
BW0\_6
AW4\_1
AW4\_2
AW4\_3
AW4\_4
AW4\_5
AW4\_6
AW0\_1
AW0\_2
AW0\_3
AW0\_4
AW0\_5
AW0\_6

585367209663758761687020072796684206495862494479836093671921697387304241972518817184071895713005407769196744747444947436665827109671629622927000271346617726974371437719506985857005685925130373363637396977175667690365222651268486606761770197

872825236358107731263169627217

872825236358107731263169627217

872825236358107731263169627217

872825236358107731263169627217

872825236358107731263169627217

872825236358107731263169627217

872825236358107731263169627217

585367209663758761687019272796684136495862494479836090871919697387299041969518817177771895713005407769138743677444947436665827108971626622927000271220617726974371437719476984257005685925130373363637396976275667690305222651268486336759670190

5254459107423214573073434428440121634175491221601172540675154615214928334715136123813230319190

310

310

310

310

310

5254456107423145730734344284344163017549122160117254067554615214928334715132123813230319190

5254456107423145730734344284344163017549122160117254067554615214928334715132123813230319190

5254456107423145730734344284344163017549122160117254067554615214928334715132123813230319190

2

2

233218133027445192148912193111325392353311011833934

233218133027445192148912193111325392353311011833934

53661931444502582528413413651495224141443116146182236117212356217287122

53661931444502582528413413651495224141443116146182236117212356217287122

5252332812734302612512726553342716132868

5252332812734302612512726553342716132868

684

484

22

22

22

264

264

264

2

2

2

2

24

24

24

4

4

2

2

132

13

13

13

13

13

2

2

2

2

2

26572

26572

26572

26572

6572

6572

2

2

2

2

2

2

2

2

841118117316258123109

841118117316258123109

841118117316258123109

3

3

3

4

4

4

11187462583109

11187462583109

11187462583109

811279

27

27

8119

8119

3

3

3

3

3

3

45275

57

57

57

57

57

425

425

425

425

425

5423311796917925167194131811872625102194479930614474164101822439142138487843206242010

402316179691791671941228126101021647993011410164101819315153222371320616158

17419

17419

17419

17419

3433

3

3

3

3

3

3

43

43

43

406161796617916719412281221099124799301141016410181912515322271320616158

406161796617916719412281221099124799301141016410181912515322271320616158

406161796617916719412281221099124799301141016410181912515322271320616158

406161796617916719412281221099124799301141016410181912515322271320616158

23

23

23

23

23

14152591671234636434896626413852

14152591671234636434896626413852

14152591671234636434896626413852

14152591671234636434896626413852

14152591671234636434896626413852

567654955623309345203318

567654955623309345203318

567654955623309345203318

567654955623309345203318

567654955623309345203318

567654955623309345203318

889569591331672922432021177230080014936445712031906805161317513404413742369248910031911210134513341205635726967365369532824688948516286248306103433597252297145316441002636470

2822252

2822252

2822252

2822252

2822252

320169190250553092588033931035612351364489334143313027835016252943571101593122455637152335669032036337652199262235127

23059100161946053975925235130325617281910614121319229193102233329501222628824172061977861344112915611711

4086

6

6

408

408

23059100121146053975925235130325617281910614121319229193102233329501222628824172061971861344112915611711

33

33

63

63

230391008034605377592523513032561128191061412719229193102233329501222628810171861971861340112915611711

48

174522652

2303980581460537759252351193246112819106141251922919310223332671122162888171861971741340112915611711

2011514106

6

54

54

11

11

20142

14

2

20

389236

389236

22767823613345

33

3

3

3

3

2245

2245

2245

767823583

7682356

7682356

23

23

7

7

15426762

15426762

6676

6676

9422

9422

39781008438757264355296572367663011312227

39781008438757264355296572367663011312227

39781008438757264355296572367663011312227

39781008438757264355296572367663011312227

3633

3633

3633

3633

272211102

272211102

2

2

10

10

16211

16211

110

110

2

2

554269143613321951510126941091143011522712017171939342678387226852943175

136415444724

136415444724

136415444724

35

35

35

5

5

5

2

2

2

3

3

3

823232677253521448812

225819447

225819447

4356

5

36

4

5

5

2

2

3972522

3972522

38

3

8

4

4

22

2

2

2

2

25355213161326192154616109330112235310617273418159763552

32

32

25354912661323192123716109330112235310617273418159763052

25354912661323192123716109330112235310617273418159763052

3

3

35336

35336

686224

4

4

6

6

622

6

2

2

8

8

22

22

22

152311

153

153

11

11

2

2

722912233

22

22

3

3

5210

5210

2

2

293

293

642444215341273

244415341273

244415341273

6

6

2

2

4

4

938932731196597144493

938932731196597144493

383117

23364

15053

655

65

5

837493

7

8

3

3

29

20

56327231627

5631279

2711918

44

44

13156822148124491041311144

13156822148124491041311144

151

151

13156822148122981041311144

13156822148122981041311144

222

2

2

2

22

22

22

6762315

6762315

6762315

6762315

123413

123413

93413

93413

3

3

22

22

22

22

172412473326683314244565314788420171315395152333126

172412473326683314244565314788220171315395152333126

91551653724

1657

9155324

7351671452262

16

3571452262

7

1021722331939321424456331478820631539512162512

1469

7

413975

2

102179352256147821163162512

2233101816412

332

14

2

2

2

323

323

323

323

3368117738392602245662218171610

5

5

5

5

3361139272245662218171610

3361139272245662218171610

31139272245662218171610

7

3113922245662218171610

36

36

3

3

3

3

327433

327433

327433

327433

353

353

353

353

76722149141191631019516102026867222734454679395115348

53

53

53

53

19212

19212

19212

19212

76532149141191631019466102026565222734442679395115348

66

66

66

765321491411916341946102026565222734442679395115348

1018

1018

63353316316

63516316

333

2349

2349

46

46

441199

441199

669211411116461020265522273449395115332

5

66921141111646102026522273449395115332

4933253807621851318102217392914711450744348521860782107317053192753172323245690218389388447329114649153916614086891331462484832281752076752983688162811391034963575443

6

6

6

6

53915043445652853035224721513340957255143944562742393026833123

2

2

2

61925182621352471854322225274204262483

61925182621352471854322255262457

43

25

6

6182610376526

16

12

6

2

5

25

857

1921516

15

26

26

2269204

269204

2

219

2

2

19

19

3313118263528592390407374714374463330223940

3313118263528532390407374714374463330223940

2

7

72337

6

11433040

9

20

110

2

265344

113263529023819111

33

85

14

7

1840

6

6

80

80

80

3

3

3

632

632

632

632

7

7

7

7

27897963305566794617811530854214115605171307545141378739257121224541491633359461582836541170334227131033785939561052142239875496261

158

158

158

33021593316725

325

325

5337

5337

16

16

139

139

1721

1721

1681259163441423984757338225126126664576213817424511283692191646133764383100141015

74

74

228

228

12479643

12479643

168571564410239847573382211261266645762138174245112766219104213376435510014715

168571564410239847573382211261266645762138174245112766219104213376435510014715

5132

5

5

2

2

13

13

92671366

92671366

92671366

344

344

344

22

2

2

2

2

16234104069149335630276141136

34234703117211

34234703117211

191217

191217

4101532161463027614

4101532161463027614

6

6

236

236

12

12

815430406853704322232813331234267528410941213177491819

7244

7244

1617

1617

96411

96411

3

3

142265

142265

25

25

1319

1319

239

239

70113033685370432023289312326210941213176591819

70113033685370432023289312326210941213176591819

5

5

4

2

2

2

2

161025132331118118

3

3

1018

1018

1625132311118

1625132311118

10552370717291942141016971719683867900236994145816278457065351702102277115152339016852468227

8552370717291942141016971719683867900236994145816278457065351702102277115152339016852468211

8552370717291942141016971719683867900236994145816278457065351702102277115152339016852468211

216

216

394762

2

2

2

39476

39476

39476

32

32

32

32

352232

352232

352232

352232

4218351218612011235644541027412532742914365402738

3

3

3

4

4

4

421835818312011235644541027412532742914365402738

8

8

322274

322274

31211235644271027411415540

31211235644271027411415540

2

2

78

78

421535818275329212738

421535818275329212738

411820121027813299

411820121027813299

411820121027813299

411820121027813299

536

536

536

3

6

5

1237433353

1237433353

1237433353

5

737433353

146

146

146

146

1662052102478716976157726335611293220479102472681312311033101444150881517991103324165106127366079296307161117482122156715027418005622559376728879144

64595713368748631152614918339855352152

255

255

8

8

6459571336548626102614918339855352152

6459571336548626102614918339855352152

4627679643438766

38

38

627964336

627964336

467446

46744

6

2310

2310

2310

735149572147622622560372272616176506264941355319438021566323

1565

1565

726115

726115

7171451476226167323722726161765062649413553194380566323

7171451476226167323722726161765062649413553194380566323

18358236

18358236

5

5

222

22

22

2

2

3815364011152113533

3815364011152113533

1111

2

3815362911151833

17

5384979781977695371311122321398644722010564722191388143113117746833201241038810279296288161117482082155614617817121701893361285571144

6

6

125578557571699518131112232085564472207325672191388143159177464227212491739537245230877848171176569317817011241233361155542144

125578557571699518131112232085564472207325672191388143159177464227212491739537245230877848171176569317817011241233361155542144

19165121513

1513

512

19

16

4034203731

4034203731

1477811

7711

148

26

26

41152662619172639572414842112454318534629

41152662619261957241304211244431533329

8

17

20181013

66

66

1250

12

12

50

50

11

11

11

51211554822781629553331209651024271012074919337329659691780253640102110029081612631132163011785634890562395256171821525885284881204511832101483713819743145241572725699

13162291331895891237762395452292

39

39

39

39

2

2

2

2

3

3

3

3

811188126545837439

811188126545837439

811188126545837439

811188126545837439

5511563444362562292

244625322

4

4

24625322

24625322

4

4

4

53756343392

53756343392

53756343392

25271114146831872632924538347221147711812651105862935649568350139933352701582617164961532615071231333444438673161385332

230143621661378291419382952163127128510413125029

230143621661378291419382952163127128510413125029

230143621661378291419382951652527128413125029

1238

21710121147385913124018

1342421661378181029127162712841011

516510

516510

2327184146831872632924524311221147711812651103696798648768321125742972701292115004651505495071151283344037373149335303

162615230

162615230

162615230

1517253825216212450136244425612377261731241699141716517583

1517253825216212450136244425612377261731241699141716517583

1517253825216212450136244425612377261731241699141716517583

11145221513215106881031056641216014296141152136412

11145221513195106881031056641216014296141152136412

61237

483121343

111451871075108810310566412135112961449136412

2

2

610231119144820637191622761511122014751089311142069169

176719166313884316117

176719166313884316117

2317812849

4589

19121284

6106743311352

6106743311352

5271076981276759511143

56

27107698127759511143

624252761913012446115510108771091114360771442751833132928112221380415140432227847327640180984144199

45

45

5710742323822105335

5710742323822105335

2

2

33755748271573134514311522

33755748271573134514311522

18

18

62425276191307445310989771098740538651404512131258681522444061305302076368274267993611749

62425276191307445310989771098740538651404512131258681522444061305302076368274267993611749

2

2

32121531012396117110565411379

32121531012396117110565411379

3378

3378

4417153571254546285682067474242133860466800655727801169819556612665786149350893446910721934046404343824210486506469481118891164997783655371719135925433825361

67179661158212134668663456842297395170515814466810822042391814098314845539933381319834748722029208892568781281474922027225268

67179661158212134668663456842297395170515814466810822042391814098314845539933381319834748722029208892568781281474922027225268

12603210141264133961925781143634272742381412789145072736

12603210141264133961925781143634272742381412789145072736

55179665582893466856345684229739515639588053869214404893140905137412336291741124347249792918189247964128974219323625268

55179665582893466856345684229739515639588053869214404893140905137412336291741124347249792918189247964128974219323625268

2715166221

20712621

8

8

5768

5768

15755

15755

7842

4

4

384

384

2

2

4

4

4

4

613

613

613

613

4431171349521

4431171349521

4431171349521

4431171349521

5

5

5

5

14355

14355

233

233

62

62

65

65

22251056049313580941244133455633673671931944912244553221435101397398679830058127386103416

162765434345521684

6534

6534

16274434552168

16274434552168

233

233

233

5823560493130744118356277523302300193188451184453216414101137326679029954127386103416

4235343

4235343

5823560493130741183542775233023001918845118445321641410113732667902995412738173013

5823560493130741183542775233023001918845118445321641410113732667902995412738173013

55768406167340266610

55768406167340266610

55768406167340266610

3633391523241352319852722938

3633391523241352319852722938

363339535

363339535

15232413231985272293

15232413231985272293

157

157

7

7

15

15

32462043234761029495124175

62042469173

62042469173

62042469173

3

3

3

7695342

7695342

7695342

321334249

4

4

616

616

31533233

31533233

14949028137946183013062731282014290357245963401264114422535447578249143298432136556674669121519217738134313441922172835

3546

2

2

33

33

46

46

1317810912599901335

1317810912599901335

1317810912599901335

222817263756

222817263756

222817263756

146610956391752131866111685414810

18

18

56

56

11

11

14664

14664

563917521663414

563917521663414

61091313810

61091313810

135477275370461871306273128514277106754889125611442253544546554914317843213655671564149512142871235441887172825

52

52

33

33

34256211126239743371272

34256211126239743371272

45

45

3930162510167828125

3930162510167828125

10145226937045987130227312851427764453264123851410535443255440143178425136556715641495890250985429815172825

10145226937045987130227312851427764453264123851410535443255440143178425136556715641495890250985429815172825

6

6

6

714

4

4

710

710

231601033116565704187

231601033116565704187

231601033116565704187

753140112

753140112

1061

1061

340

340

7512

7512

3

3

348372744

3372

332

332

7

7

48744

48744

48744

108255

108255

108255

108255

4

4

4

4

26527

26527

26527

26527

26527

4522

4522

4522

4522

4522

316233597

23544

3

3

3

2544

2544

2544

316353

31635

31635

31635

3

3

3

261375135268921319120332640692424621453016133047026165682173350261246242022

10813319

10813319

10813319

10813319

261936109232029420232124171617352818915622

261936109232029420232124171617352818915622

261936109232029420232124171617352818915622

261936109232029420232124171617352818915622

36

36

36

36

2416957211

2416957211

57

57

1910911

1910911

562

2

56

10212206335111621131313378188525187261312

1210

1210

1210

103551116813137378188525187137

5111681313737818858187137

11135

5168137378188818137

7

35

35

1017

1017

5

5

5

10

10

10

25328136165

135

135

25

25

6

6

2816

2816

3

3

31291267

6

6

6

3129127

2

2

3129107

312910

7

4

4

4

4

2

2

2

2

3513875665427333152412242

3513875665427333152412242

3513875665427333152412242

35872

655248

766542233315424

12143247402974737225512818780133711543344751109271183319121443791370965624270113778104166116354846133329512742337537

22

22

22

22

22

12141247402974737025512748777133711543344751109271183319121443791327965624270113778104166116354846133329512742337537

12141247402974237025512748777133711543344751106271183319121443788327965624270113778104166116354846133329512742337537

12141247402974237025512748777133711543324751106271183319121443788254965624270113778104166116354846133329512742337537

12141247402974237025512748777133711543324751106271183319121443788254965624270113778104166116354846133329512742337537

12141247402974237025512748777133711543324751106271183319121443788254965624270113778104166116354846133329512742337537

273

2

2

73

73

533

533

5

5

33

33

43

43

43

43

43

73

73

73

73

73

2

2

2

2

2

2

222

222

222

222

222

222

321971322222132341425

42

42

42

42

42

21559

21559

21559

21559

21559

2

2

2

2

2

38

38

38

38

38

2

2

2

2

2

75222212644125

722

722

722

722

522212642125

522358223

3

3

52225022

52225022

108

108

2906102

2906102

2906102

2

2

2

2

2

2

56

56

56

56

56

56

160

160

160

160

160

160

5867243022361599218707202119160919668382377113062335235927789162843630760183545180193616952145611212322180425783322221661416566107921510531519014145116176084

2

2

2

2

2

586524284234159841869520211415541966838235051290733523353778916283813068618354518019356695214561972314171422382542221521416358107921510524517414145116056072

555518238234159701868419911415401966829234971289933523353778916283813068218354518019356655214441972314171422382252221521416158107921510478517414145116056072

202474

202474

202474

562

562

562

2624827262317334257462046515432545522414835

52422112006543

52422112006543

2624327262317313634204512545522414835

2624327262317313634204512545522414835

3310277013471692685664141153935946645176295213681851125266766

31027701347112266259141153935946451262941748181125266766

143414225111694125

871117

278421721868306272438724254461513

12174

223854

54

3940691473663505156272525684101969

519259210

7

9115

555107

357254565011625

357254565011625

21123261449218955961080152523222821110781180321681910481614321623713751181566740712459271191

21123261449218955961080152523222821110781180321681910481614321623713751181566740712459271191

2510217124428461159423397559623011

11

36

7

27535

191129

52

95559956822926917145873449

444

12

16913242007561227108018811021051582224319320338852115320326210131

14

11289086913

3

3523

3

51394712

112151311121362899122524

912165070988110123719521114836

35

41512127173119421414119412331636222386125582552226138632080222340993621191615415311402132205163370174311712354106368831139879244813249110635613

27298345210418204365345395323307010123416

8

478414122

232203042104182043653453953193062103216

41512921661161413796194123116350219401254322921800386319167205048531711216131931140212420516336997401151235495368761139867244813237110295597

41512921661161413796194123116350219401254322921800386319167205048531711216131931140212420516336997401151235495368761139867244813237110295597

2104

2104

33302815625109112979456821301172

33302815625109112979456821301172

21182274020121096843648516825274011054233011555109

35

35

2118224402012109684364851682274011054233011555109

5614

296234

17190278722023323756212825423151720

2

210120567449829821955

141320101047201928573319

9448146

22468278253751872

2456445

2456445

212278253311372

2122782531137

3

2

745573213841176311317621063228416604813164955414672616842517334398202318122939102530129152428214158

8326733426

8326733426

8547992411565242404488951371072107013276467432131599

8547992411565242404488951371072107013276467432131599

486326325104441013863954205523541252841123711

486326325104441013863954205523541252841123711

1816610

1816610

74536171251221012813161436231225172524751018533108263215241022

74536171251221012813161436231225172524751018533108263215241022

7615795676859371185338

7615795676859371185338

313267137

8

35267137

49

49

2

2

2

3311398

3311398

6

6

113

113

2

2

3

3

3

3

9

9

106425148121846

1058946

10

10

5

5

3

3

8646

8646

64214129

145

145

54

54

7

7

642

642

244

244

24

24

4

4

46632

3

3

3

6

6

6

462

2

2

46

46

1865935

1865935

1865935

1865935

1865935

2212462661552395574592489347514282161212

2212462661552395574592489347514282161212

2212462661552395574592489347514282161212

2212462661552395574592489347514282161212

2212462661552395574592489347514282161212

2923211203110672496738133316147141173921172751541027434612243048012963

2923211173110672496738133316147141173921172751541027434612243048012963

2923211173110672496738133316147141173921172751541027434612243048012963

2923211173110672496738133316147141173921172751027434612243048012663

8710

8710

2923211093110672496738133316147141173921172751027434612242348012653

2923211093110672496738133316147141173921172751027434612242348012653

1543

1543

1543

3

3

3

3

3

996471569264664519232127311

4422

4422

4422

4422

4422

9871362436376321237

362312

362312

362

362

231

231

5432874

2

2

2

5432872

5432872

5432872

3824245834

3824245834

34

34

3334

3334

22455

22455

3

3

2

2

621323

621323

2

2

62133

62133

2522

2522

2522

2522

2522

2

2

2

2

2

3

3

3

3

3

84244211605

26812

26812

476

476

2

2

2052

2052

84424793

84424793

84424793

84424793

4

4

4

4

27

27

27

27

27

27

7

7

7

7

7

7

56973700236256368311680875355146489608246165446617365434632067733462512727448820452816961670445528913531738133721412605963768456107036960760664885021660408685826167760242503375236675385052060584495795443570947539594994147777314305388330137

563026490358097658306601728721272896047160662460466333275116710717357852848236175656856770149346391453015136714272280562737272624820360149658674927860202678916089984141492542625469727626587824761544698108341255921603472539416114408062

463633495

463633495

65

65

46333445

46333445

1195654218423177637122310266227413885232323

1195654218423177637122310266227413885232323

1195654218423177637122310266227413885232323

1195654218423177637122310266227413885232323

99174471117382230

99174471117382230

99174471117382230

99174471117382230

772136126103573822518621583170179831231234417258443316341364204894036188554423191613

3

3

3

70322252558826732669525037343061076982259695

492711575867226251363724306106158209560

492711575867226251363724306106158209560

5

5

118235032

233

2

50

8

11

21117722735077

21117722735077

4283434

4283434

4

4

1024

1024

7021041045107377171789114411083123123391475814261291134174793275206528516241613

702102915102377171669114411083104123391475814261241134174793085203528516241613

16

3676215142318345

315666

3523777812121922621123510

37

9301831

3412776

25317421194184653742147101551536216

8872

10

68349511121742911163265747204

685104123164191143239613

7

28

19935682155252622663681023074588

2256153

614

285125737109102

5

31

5

5

193

193

19

19

13

13

12

12

5

5

2

2

134192228213906722621483151710113635034010986445261815204672018771741502173301811144557795683313218021273232919394801102825603791689365450345

93349661575232067758187171251067117314551644155702443

157770786

157770786

2

2

36673121255814515631115

36673121255814515631115

93496651614587310911735164457728

93496651614587310911735164457728

12519192517381666262883151710112134333510754439261744453862018771661495173131691093557728682212517719873232919389751086821599790184295426302

38

38

257189191676040521349591522404404453537126091411511679311801010282323

257189191676040521349591522404404453537126091411511679311801010282323

6331551027057223

6331551027057223

17

17

123

123

1251919109244973820108981062339335100844342617416310018163716210551731316464232817109175837321291938064243736407789174267385251

1251919109244973820108981062339335100844342617416310018163716210551731316464232817109175837321291938064243736407789174267385251

2

2

88193223174122732682139222558

88193223174122732682139222558

53944173388748916711

53944173388748916711

43538608423786608495303

43538608423786608495303

43538608423786608495303

43538608423786608495303

712

712

712

712

274

274

274

274

2213

2213

2213

2213

363321988609717271101056013110510442012944135754841485694835708

3454360671727591056065105284201448765484144469435708

32

32

418

418

32

32

60675995605796284201448765462144468860

60675995605796284201448765462144468860

451117278854635108

451117278854635108

362814453051667629481418

362814453051667629481418

362814453051667629481418

52

52

5

5

2

2

6

6

6

6

5536064739579066576965864282642607360184606484602961982593566798159198118475621617868226699258375132821384871359190026247925727308695993565588488895962567790540608134146164042473242470756912415944149092961118113104447668966104297356

54505630495590563287641142801826016569705907745061611925515634761541079694602715665641446968753811233012851655731265652377252162634159298652114187659536674345400279831444239454672024201450011512353832745102408698325318563100076947

36264

36264

79454064827505248742477217282164732680527528201672774237041671331059421287664821258852105195312222542817260237472151331513222537635214393502444122415324446744887766970374421826132307985421322735002065

79454064827505248742477217282164732680527528201672774237041671331059421287664821258852105195312222542817260237472151331513222537635214393502444122415324446744887766970374421826132307985421322735002065

128933914232373941341489192412130101685024121896332108134629821162132249157216252937976230359440630036459616801769444104215101734869348113676033291122502362824371137371504

128933914232373941341489192412130101685024121896332108134629821162132249157216252937976230359440630036459616801769444104215101734869348113676033291122502362824371137371504

4

4

3018937247

3018937247

2

2

452711901024168346743517810719943628241194191472628342273357410149457756352541508055380482162280970710269504146250348872248318686396835162535897226422790820117470076061765384031507736895372277891358650842722739519961364376

8048622150981317479321439440584820311974826

469040104413663113481552118310054151177184511045016018874160734145116356

2189515784208573062530824931377812563817706135062122213973297113050657531242129773155642093209562216727484734254215512100616980222554343219706158331947819439440871111346368831430630463205242171184589940572506416053803977

1231444575020654020460225128981004020863272147874543137681688719554313840112591605342

226713991305228897137170331404236333682616226201338481149910647

10052

120

209403087298637404029140615012542153212206231281238171745108337781699228235529572731313717571873585813601242171417506963760787720415168315251112675255027024179249510329664594254

3735212178730198112342207646241011434959810610172235161125192245625386731915515112

2262121730868766416132120254913992154

2120

22212173086876641613254913992154

24

35987198111536127639249513785981064411516925191705625377718063108

19126424619886216180

3613

3287148398359798

14282512

39511151219105984411516891380810

6257

17711773246955

206212035186627272537402330228583236972876336608275

6

6

46814885

46814885

10405

10405

1165020164

4852088

1116576

68281758168353

68281758168353

20621103518662721173723302285832365827

2062110351866272117372330228583235827

6

5868133118

5868133118

5868133118

7841582187224071623144342573149693562395329231892882316393522329017847855517457799864974400637368670789306451511687221591600711914652227921392411851403398264

1066125171102091976268319420224149979754

683174

1252020224149979754

6

1061711020919762

7841582187224071623144342573149693562289328630692882316393522329017847805517457799693874191637349594589306451511687214760591311714652205921388311802306301210

2954716161554788411469941119422654855

34877411885244264903352781136051177621787167131715012111110770689691165567608531492069341230510103782050512432

1012192019112523

1087714177825

381286851351967219520395226132482117962826439025283150316829432

20

48544242412355

56541542212515

101112

9262611715

7281458167621291400172523969726002120217832299261221819475115625305585163432813372263999466255414781108209614851261921371158172200337328406434241243135

114836

967106437515234255625231223756912611015293831198117124534483423704155570359285281331213163202928

56710643753954725231029948152925231131945344826531091837696107

56710643753954725231029948152925231131945344826531091837696107

173422127342207682516030722792840825491801181622

173422127342207682516030722792840825491801181622

521136763731985246530129191108253671372105141274986

521136763731985246530129191108253671372105141274986

410310332223712

410332223712

103

2254338316802516096234

2254338316802516096234

234914287411841061383218645522131613192029302209023843423190757471042616112583849203685104315363582379666142652

234914287411841061383218645522131613192029302209023843423190757471042616112583849203685104315363582379666142652

234914287411841061383218645522131613192029302209023843423190757471042616112583849203685104315363582379666142652

234914287411841061383218645522131613192029302209023843423190757471042616112583849203685104315363582379666142652

234914287411841061383218645522131613192029302209023843423190757471042616112583849203685104315363582379666142652

123315156561113638114613023442037617242561953532171714558161261167527268

9279132488917371145330233920276172561941276849114272028

57130128433914248661725619156681575272028

17554306

17554306

3111653914186131238271428

3111653914186131238271428

5421214368612126133668157

5421214368612126133668157

2

2

42092365173229222333202726213439

193

193

3849

3849

42092172172425222333202723213430

42092172172425222333202723213430

366248221910851024125511914591612116487240

366248221910851024125511914591612116487240

366248221910851024125511914591612116487240

8513129

2

366241761085102455991451612116487240

3

3

3

3

3

148813212632

148813212632

148813212632

84

84

14413212632

14413212632

63650384437229719402353518103350992571262831693150726673171335652564999625018232185282048166332521004164162210360759587520266076240570315289541278822877170318559648600443491428325518181433580219155

104411654

11654

11654

11654

1044

1044

1044

2131919295026966621162299915948394562172428434211217656941477707430

2131919295026966621162299915948394562172428434211217656941477707430

2131919295026945559162099615868394487152206434211217656941477705424

2131919295026945559162099615868394487152206434211217656941477705424

216223875222202

216223875222202

6

6

39665420348463584061800223411073112218314220328025117114178432743928191497182916155988912842118261468713

39665420348463584061800223411073112218314220328025117114178432743928191497182916155988912842118261468713

39665420348463584061800223411073112218314220328025117114178432743928191497182916155988912842118261468713

39665420348463584061800223411073112218314220328025117114178432743928191497182916155988912842118261468713

434282410282101553167858364741221410929213053

434282410282101553167858364741221410929213053

432824102794553167375164629449529211251

432824102794553167375164629449529211251

42557483212281014182

42557483212281014182

15921828854435499833502408804586924771904015328125538926885215966194622442501096326256521633417118751272663282886817712358226171085105081175921564728163155648609474

54

5

5

4

4

19833018451910538487719822293417185738

7615159961099

7615159961099

2591071320115

2591071320115

17768

17768

721192723215407142818

721192723215407142818

21354181011465614143931196

21354181011465614143931196

352206

352206

352206

15921828854435497773472408804583924591903970326224538396835215966186221572501096319254621633417118751272663282064527712353226171085103081135920962628163155630552436

15921828854075497373472408804583924591903970325624538316835215966186221572501096319254421589417118751272603282044527712346226171085103081135920961428163155630552411

15921828854075497373472408804583924591903970325624538316835215966186221572501096319254421589417118751272603282044527712346226171085103081135920961428163155630552411

5

5

4

4

16

16

30

30

206212

206212

682447

682447

620

620

1501536917413035403673394269692275461151015368079596067448354190457994421167264665173279381331041473359652458836981382628511949191245367111836245

94239

94239

94239

13779591016443964528424822301011633533201258326549413519372099523454943382824692442521091161767423652106933661831042170660793169

3

3

2254317412030762

2254317412030762

11151452064788415101367118982327207336474364206117205510128771023761679565

11151452064788415101367118982327207336474364206117205510128771023761679565

6

6

80164941181031794118835312157746312728

80164941181031794118835312157746312728

5548348044324423324266522445444132532821402624252043213032255725283695377781815

5548348044324423324266522445444132532821402624252043213032255725283695377781815

2210346326381532217212721

2210346326381532217212721

61111326539433470272382210563843155817933423058645482082

61111326539433470272382210563843155817933423058645482082

461310

461310

5522537216726214014

5522537216726214014

11342266

11342266

1322365

1322365

2112633212261626475513129612813612102174

2112633212261626475513129612813612102174

671521544355566714221255317

671521544355566714221255317

16431972856319201033161737312721409338545330463834242304259118

16431972856319201033161737312721409338545330463834242304259118

55216201278954497362814

55216201278954497362814

102825632282875702282131916131738814291106

102825632282875702282131916131738814291106

2129451808221654547653079209134346242

2129451808221654547653079209134346242

2129451808221654547653079209134346242

2

2

2

202131950343517955731257160269521020725322132665511542835629875939612933660

202131950343517955731257160269521020725322132665511542835629875939612933660

202131950343517955731257160269521020725322132665511542835629875939612933660

402030414149252711368121232917511458

4020321413335612123715324

4020321413335612123715324

24473310

24473310

272121491114812216282824

272121491114812216282824

1354852472877266111164158588421625846143759922521964219542662571326234976142547131933994150433923138414707673284036832107

712453318149025811856699911851269237234111320924211255551051222197852521

712453318149025811856699911851269237234111320924211255551051222197852521

282616161514821196810342652149551331989573694454705524357

282616161514821196810342652149551331989573694454705524357

47616915824472446920627846456163641206092754151122294146

47616915824472446920627846456163641206092754151122294146

46

46

103911237753

103911237753

3685663541528

3685663541528

42021162184759242618478

42021162184759242618478

34324134280499372284774233835531713169757114111896262175807467

34324134280499372284774233835531713169757114111896262175807467

5625171614104639223588

5625171614104639223588

8192222212042463118561099666

8192222212042463118561099666

837210151390150344145445314392855046117169784412444114223211721136311957018990111373798380

837210151390150344145445314392855046117169784412444114223211721136311957018990111373798380

9612292324725113811514912685551835367445889632951613213812361

9612292324725113811514912685551835367445889632951613213812361

962

962

962

653

653

653

101111230210701015362411559948590492428456336283121111503310811319231591392359241286587711314596523361413510

101111230210701015362411559948590492428456336283121111503310811319231591392359241286587711314596523361413510

101111230210701015362411559948590492428456336283121111503310811319231591392359241286587711314596523361413510

101111230210701015362411559948590492428456336283121111503310811319231591392359241286587711314596523361413510

922459335412601065312228842041113938135663260116022125771295221795324582116719382751255226691211282667234150851127130819321413018969752867994117201676543374

2131383

2131383

2131383

5366231775

35

35

536323177

17

5363237

82244074581031651031865565901710011122331141283321342026055260202

10544326

10544326

64081743487113

64081743487113

263512988868895435128

26351264415128

9888849520

82123568112951122734141245415183859

12

8

242

82123568112951122724141245415183445

16242752213

1624213

2752

201817

201817

203181543

203181543

1933

1933

5

5

31663178

31663178

134615856162537192697105127335117

46156154537192697103127335117

1385820

5824

5824

21503523532512973277858741615051133105

21503523532512973277858741615051133105

292419334710651010670375912111156558537415821335572621791044114110718612255451916346201820999112078817921490815275133823611491571231

5

5

2525334421

2525334421

71465751084178727931796210513842722479181117349164265230302109

1354

1027296355525

4

204061811717787847798403497527022

45556721569278222202402337711179101044811662

14111313194842296147

106

14

6

7611344354

69

13

13

2412332810419952123205938017543346343

2412332810419952123205938017543346343

536352

536352

1451915603294912112153932821311423239114677824992215111665533201820973905414521490725115061053611261243117

1451915603294912112153932821311423239114677824992215111665533201820973905414521490725115061053611261243117

2610

2610

3

3

6

6

3

3

932756669

932756669

283768686304652709332223410658

28376868630465270933223410658

2726730240744710

281042686231653019321949948

2

2

52146

52146

52146

113322052633

113322052633

113322052633

454171582207141453966238064043153073332519522812932129428361504611114154037602391009548

454171582207141453966238064043153073332519522812932129428361504611114154037602391009548

454171582207141453966238064043153073332519522812932129428361504611114154037602391009548

593284922161850214265261913899611401169605704362534732164276863208631881743238910112512952122256082313

4

4

4814234

48234

14

23

23

845525703122415

845525703122415

296650617124821468244318350013265112299111

22682418349391337441

15

7

9405061712482131441324922170

153484

19286207

196207

28

8

8

2

2

42

42

2935735103

2935735103

6722637261633449627630174

12732

5522630261333449627628174

8395241310967046

8395241310967046

8

8

6733

6733

592923711671295210945042124587912269937241734745219628658631521207517131085511041222107844031883

592923711671295210945042124587912269937241734745219628658631521207517131085511041222107844031883

3123041

3123041

31117102116924628057526

31117102116924628057526

3

3

244237485171693027

244237485171693027

85222240592079

5222124

167

8240479

17531044117495617

17531044117495617

3271325476

9

2371325476

58013116183112853153378822

58013116183112853153378822

58013116183112853153378822

18539163273171441441664181923765510811941912071121361808

18539163273171441441664181923765510811941912071121361808

18539163273171441441664181923765510811941912071121361808

18539163273171441441664181923765510811941912071121361808

184154501931398875104504112331081316021284765270678466263924744004933124471621535113602012249175717279690019496102212163565011036431324069112775894

66116481814142420

66116481814142420

66116481814142420

184154501931398875104504112331080715961284754270678465663924744004933124471621531113602012249175717271688219496102212163565011022431324055112535874

15918223

15918223

68592

68592

74525418102

74525418102

635535172550

635535172550

71160544

71160544

223

223

94371042196355224583335

94371042196355224583335

49

49

548692791123

548692791123

7959

7959

3

3

4210334819311733

4210334819311733

22197653432338768

22197653432338768

3232445834755146836211823

3232445834755146836211823

433986119478817451

433986119478817451

7871449911978101111181169841049111013566

7871449911978101111181169841049111013566

44109151812114175115115476231246117819

44109151812114175115115476231246117819

3595

3595

52211615332581701211712162442243828313254

52211615332581701211712162442243828313254

1436623620

1436623620

1510141125112531151510133014410187152099422617228152213581512551636

1510141125112531151510133014410187152099422617228152213581512551636

7762266581860784529713714363242540419824291035103977621361648252854286810618050899910141478927997425402604101954487

7762266581860784529713714363242540419824291035103977621361648252854286810618050899910141478927997425402604101954487

2352

2352

181225858

181225858

35137

35137

11172816101512378232054686613541435262742125516110122552156535

11172816101512378232054686613541435262742125516110122552156535

24135545434426

24135545434426

212646517112

212646517112

271810181511172271312141018191496111415

271810181511172271312141018191496111415

6373

6373

4247826941035773139508606104472273622526620711520465

4247826941035773139508606104472273622526620711520465

5252816702664128110238284118375260

5252816702664128110238284118375260

4

4

10127113616

10127113616

22723

22723

7

7

3623

3623

58286576493692318819551313

58286576493692318819551313

11112675917854144871461110231041192510152915981210313593379

11112675917854144871461110231041192510152915981210313593379

332

332

271025206

271025206

1335225436941198

1335225436941198

2

2

2

2

2

2

2

2

52972063221228

2

2

2

2

2

52972043221228

433

433

3

3

43

43

54212

54212

54212

54212

25717228

25717228

25717228

25717228

76523

76523

76523

76523

76523

76523

44040314235

44040314235

44040314235

44040314235

44040314235

44040314235

1227427451118182322312426331922837

43

4

4

4

4

3

3

3

3

507522099211

477521536211

77311

24

24

5737

5737

2

2

2

40215362

40215362

40215362

3427

3

3

3

427

427

427

136

136

136

136

21618254914223714723

7844102317

2323

2323

2323

554410314

554410314

554410314

242526

242526

242526

242526

2765434147

2765434147

2765434147

2765434147

338

338

338

338

338

82748

82748

82748

82748

82748

2

2

2

2

2

2

2

2

2

2

925387823

925387823

925387823

925387823

925387823

476883

476883

476883

476883

476883

476883

881817726712161161212282641647

3

3

3

3

3

8452051216114121023

8452051216114121023

8452051216114121023

8452051216114121023

8452051216114121023

89

89

89

89

89

644226628

644226628

644226628

644222

644222

66

66

8

8

2246

2246

2246

2246

2246

688336252544

688336252544

688336252544

63

63

625334882544

625334882544

137

137

3

3

3

3

3

28882256164584518352032291

28882256164584518352032291

28882256164584518352032291

28492240164575518352032280

9226163532194

9226163532194

28403445755182086

28403445755182086

3916911

2

2

9

9

2816911

2816911

2

2

2

2

2

2

2

2

2

2

2

2
